# Beyond the COVID-19 pandemic: ‘Learning the hard way’ – adapting long-term IAPT service provision using lessons from past outbreaks

**DOI:** 10.1017/S1754470X20000379

**Published:** 2020-08-18

**Authors:** Lilian Skilbeck, Christopher Spanton, Ian Roylance

**Affiliations:** East London NHS Foundation Trust, Newham Talking Therapies, Vicarage Lane Health Centre, 10 Vicarage Lane, Stratford, London E15 4ES, UK

**Keywords:** IAPT, outbreak, pandemic, responsiveness

## Abstract

Infectious disease outbreaks have occurred sporadically over the centuries. The most significant ones of this century, as reported by the World Health Organization, include the EVD epidemic, SARS pandemic, Swine Flu pandemic and MERS pandemic. The long-term mental health consequences of outbreaks are as profound as physical ones and can last for years post-outbreak. This highlights the need for enhancing the preparedness of pragmatic mental health service provision. Due to its magnitude, the novel COVID-19 pandemic has proven to be the most impactful. Compared with previous outbreaks, COVID-19 has also occurred at higher rates in frontline staff in addition to patients. As COVID-19 is more contagious than earlier outbreaks, there is a need to identify infected people quickly and isolate them and their contacts. This is the current context in which mental health services including IAPT have had to operate. Evidently, Improving Access to Psychological Therapies (IAPT) services are a major mental health service provider in the UK that have demonstrated variability in their response to COVID-19. While some IAPT services quickly adapted their existing strengths and resources (e.g. remote working), other services were less prepared. To date, there are no clear unitary guidelines on how IAPT services can use their pre-existing resources to respond to the long-term effects of outbreaks. In light of this, the current paper aims to reflect on the lessons learned from past outbreaks in order to consider how an enhanced remit of IAPT might integrate with other services to meet the long-term needs of patients and staff affected by COVID-19.

**Key learning aims:**

(1) To understand the development of IAPT within the NHS mental health services.
(2) To understand the nature of past outbreaks and COVID-19.
(3) To reflect on lessons from past outbreaks in order to understand how IAPT can respond to the long-term effects of COVID-19.

## Introduction

Since its inception in 2008, the Improving Access to Psychological Therapies (IAPT) programme has evolved and developed treatment for mood and anxiety disorders. It has provided a responsive service to meet the diverse needs of the communities it serves. The emergence of Coronavirus Disease 2019 (COVID-19) currently presents the need for a new response from IAPT. The main questions for reflection are: how does COVID-19 compare with previous outbreaks? How have mental health responses developed from previous outbreaks? What needs to be developed further in response to COVID-19? In response to these questions, the current paper aims to reflect on lessons from past outbreaks in order to consider how IAPT can respond to the long-term effects of COVID-19. This paper will discuss the development and responsive role of IAPT within the NHS mental health services. It will also discuss the mental health impact of outbreaks and COVID-19. The paper will then reflect on lessons from past outbreaks and IAPT implementation. It will then conclude by considering the limitations and key practice points.

Literature searches were conducted using MEDLINE, PubMed, Embase, PsycINFO and Web of Science to identify peer-reviewed articles. Reliable ‘grey literature’ (e.g. news reports) was also included, given the current insufficiency of research on COVID-19. Search terms included: Coronavirus, COVID-19, IAPT, outbreak, pandemic.

### The development and role of IAPT within the NHS mental health services

The IAPT service has evolved since 2008 and aims to offer improved access to psychological therapies for depression and anxiety disorders (Department of Health, 2008). The IAPT model and initiative is to offer talking therapy via a stepped-care model between low-intensity (e.g. guided self-help) and high-intensity interventions (e.g. CBT) which also link into secondary care services. These interventions are evidence based and recommended by the National Institute for Health and Care Excellence (NICE). The IAPT services are commissioned by the NHS as part of primary care. Over the years, IAPT has responded to community need and has evolved its services to encompass cultural adaptation. In line with NHS long-term plans, IAPT has also developed its integrated physical and mental health service provision for long-term conditions such as diabetes (NHS, 2019). Service provision by IAPT has also widened multidisciplinary team (MDT) working with GPs, community services and research institutions. It has also developed its Children and Young People (CYP) service provision. The current community need is responsiveness to the mental health impact of COVID-19. This paper will focus on the adult IAPT service provision.

### Overview of significant past outbreaks and the COVID-19 pandemic

Infectious disease outbreaks have occurred throughout the centuries and they are predicted to continue to emerge. As a result, there have been global efforts to mitigate their physical health and economic impact (World Health Organization, 2014). In epidemiology, an outbreak occurs when an infectious agent such as a virus or bacterium affects more cases than expected over a given period (Porta, 2014). This can range from small localised communities or countries (epidemic), or thousands of people across continents (pandemic). According to the World Health Organization (WHO), examples of the most significant outbreaks of the centuries include the Spanish Flu (Influenza H1N1 virus) pandemic in 1918–1919, the Ebola Virus Disease (EVD) outbreak in 2013–2016, the ongoing human immunodeficiency virus (HIV) pandemic since 1980, the Swine Flu (Influenza H1N1) pandemic in 2009–2010, the Middle East Respiratory Syndrome coronavirus (MERS-CoV) outbreak in 2012–2015 and the Severe Acute Respiratory Syndrome Coronavirus-1 (SARS-CoV-1) pandemic in 2003. The most recent example is the recent emergence of the novel COVID-19, caused by SARS-CoV-2. This virus apparently emerged in China in 2019 and has rapidly spread globally into a pandemic. A major concern with COVID-19 is that it is highly contagious with high case fatality rates.

### Outbreak life-cycle and psychological impact

**Figure 1.**
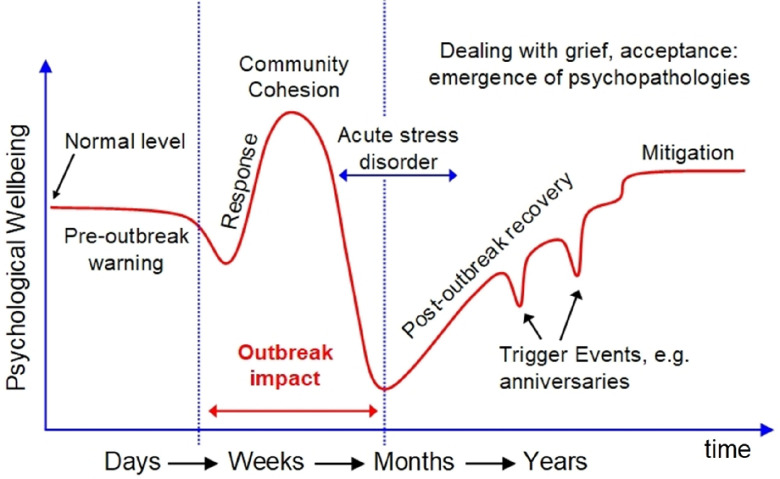
Outbreak life-cycle and long-term psychological effects. Adapted from CDC (2006) and SAMHSA (2015).

In parallel to physical disease, outbreaks can result in psychological distress, undesirable behavioural responses and functional impairment (Morganstein *et al*., 2017; Person *et al*., 2004; Shultz *et al*., 2016). Outbreak life-cycles present in three phases: pre-outbreak, peri-outbreak and post-outbreak. Psychological reactions at these different stages also vary. Most of these responses are considered normal reactions. However, these responses may develop into mental health difficulties in line with various diagnoses such as severity, impairment, trajectory of symptoms and duration (Landesman, 2005). Long-term symptoms can include pathological grief, depression, anxiety, post-traumatic stress disorder (PTSD) and substance abuse (Fig. 1).

**Figure 2.**
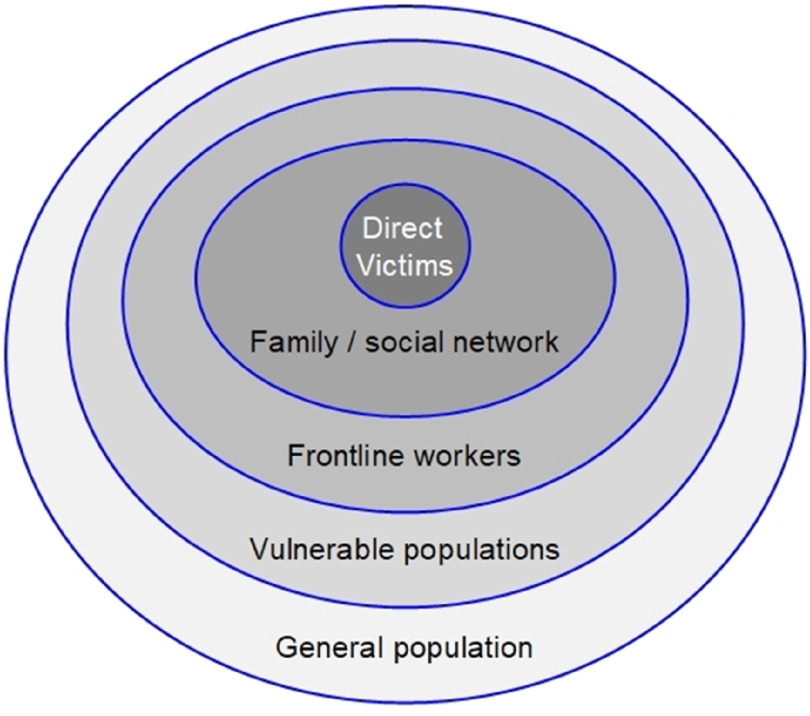
Outbreak psychological impact layers on the population. Adapted from CDC (2006) and SAMHSA (2015).

Psychological impact on the population is also determined by the level of effect depending on whether one is a direct or indirect victim of the outbreak (Fig. 2). In all these groups, symptoms can appear within days or weeks and last for months or years in relation to the outbreak life-cycle.

As demonstrated in previous outbreaks, in addition to patients, frontline workers are at risk of suffering significant impact (Fig. 2). They face challenges including fear of contagion, work-stress and contracting the infection. Resulting symptoms can range from acute stress reactions to PTSD (Walton *et al*., 2020). For example, Desclaux *et al*. (2017) reported long-term financial difficulties linked to anxiety and anger following the Ebola outbreak. Frontline healthcare workers were at higher risk of developing mental disorders and long-term consequences. Another study showed that nurses who treated SARS reported high levels of stress, anxiety and mood disorders including trauma reactions, depression, anger and somatization disorders (Chen *et al*., 2005). Several other studies have reported longer elevated distress, depression and PTSD amongst both general public and healthcare workers by up to 4 years post-pandemic. Health workers have, on average, demonstrated more severe symptoms (Lau *et al*., 2004; Lee *et al*., 2007; Mak *et al*., 2009; Wu *et al*., 2009). A study by Hawryluck *et al*. (2004) also showed a high prevalence of long-term PTSD and depression. Lee *et al*. (2018) have also reported similar findings.

In terms of the psychological impact, PTSD is a major aftermath psychopathology of disasters (Neria *et al*., 2007). The main predictors of developing PTSD include serious physical injury, imminent threat to life and high death toll. For example, the experience of being in an intensive care unit (ICU) and witnessing high death rates during an outbreak can be precursors to the development of PTSD (e.g. Murray *et al*., 2020). Furthermore, Hong *et al*. (2009) demonstrated a 44.1% prevalence rate of PTSD in a population who had recovered from SARS after a period of 4 years. The NICE (2018) guidelines have outlined recommendations for disaster planning in relation to PTSD. In line with Fig. 2, research also shows that the risk of PTSD exhibits a dose–response relationship with direct victims being at higher risk than rescue workers or the general population (Shultz *et al*., 2013). Depression is also common and portrays a similar dose–response relationship. Predictors of depression include bereavement, displacement and loneliness.

**Figure 3.**
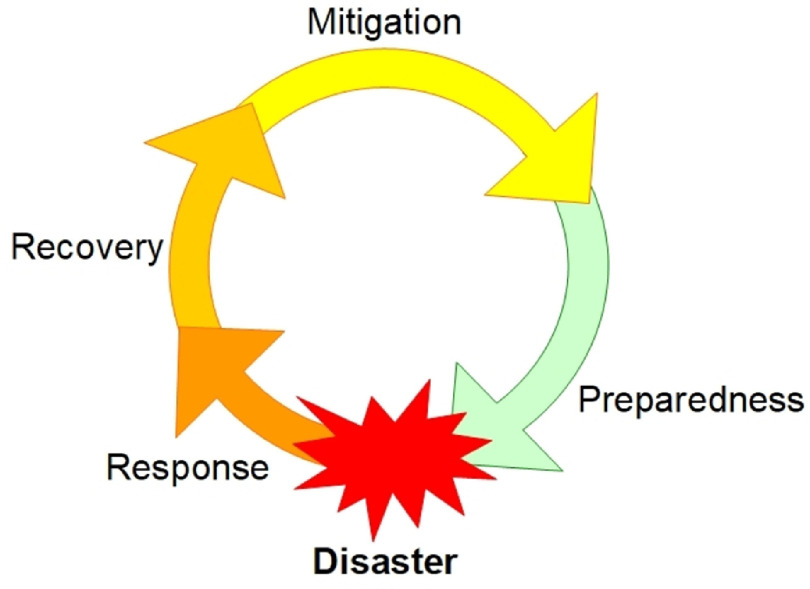
Outbreak Preparedness Cycle. Adapted from SAMHSA (2015).

The aforementioned points highlight the long-term psychological consequences of outbreaks and the need for mental health services to be prepared and responsive. The World Health Organization (2011) has defined ‘preparedness’ in terms of managing environmental health in emergencies including outbreaks. They state that ‘Emergency preparedness is a programme of long-term development activities whose goals are to strengthen the overall capacity and capability of a country to manage efficiently all types of emergency and to bring about an orderly cycle of transition from relief through recovery and back to sustainable development’ (Fig. 3). In relation to this definition, public effort has focused on physical health response. However, as pointed out by Dong and Bouyey (2020), mental health service preparedness needs to be integrated into this wider preparedness programme. Therefore, how can IAPT services reflect on lessons from past outbreaks in order to develop a pragmatic approach, integrate with other services and meet the long-term needs of patients and staff affected by COVID-19?

### Psychological impact of COVID-19 and implications for IAPT services

Unlike previous viruses, COVID-19 has shown to be more contagious with a high fatality rate. As a result, over the past months social isolation and lockdown measures have been challenging for the general population (Dong and Bouyey, 2020). In line with Fig. 2, this virus has also had a more significant impact on frontline staff in addition to patients. For example, according to a BBC news report (Haynes, 2020), NHS staff on the front line of this novel coronavirus pandemic are highly likely to develop long-term mental health symptoms. In this report consultant psychiatrist Dr Andrew Molodynski reported that the sheer numbers of mortality and helplessness was a catalyst for symptoms of anxiety, depression and trauma reactions. He also added that he was already witnessing this impact as evidenced by a number of colleagues in his hospital being off work due to mental distress.

Different demographics in the UK have been affected to different degrees by the coronavirus pandemic. In line with the impact layers (Fig. 2), specific vulnerable groups have been identified for COVID-19. For example, although children have been affected, the virus has shown a higher prevalence in adults. This has been more significant in older adults. The prevalence of COVID-19 has also been shown to be higher in Black, Asian and Minority Ethnic (BAME) groups (Ford, 2020; Holmes *et al*., 2020; PHE, 2020). People with existing mental health problems are also more prone to suffer an exacerbation of symptoms. For example, a study by Jeong *et al*. (2016) on survivors of the MERS-CoV epidemic demonstrated a positive correlation between pre-existing mental illness and post-outbreak anxiety and depression. Pre-existing long-term physical illness may also exacerbate symptoms due to the need for extra preventative measures.

In view of the above impact layers of COVID-19, current measures have focused on minimising the spread of the virus. However, a major challenge is how to get infected people to self-isolate and to list their contacts for the screen–trace–isolate measure. This is the current context in which mental health services including IAPT have had to operate. Several psychological professional bodies and services including the BPS (2020), OXCADAT (2020) and the COVID Trauma Response Group (e.g. Billings *et al*., 2020) have provided guidelines and tools for working in this context. However, there are no current unitary guidelines on how IAPT can manage the long-term impact of COVID-19. However, in line with Fig. 1, as we head towards the post-pandemic stage, there is a need for a sustained pragmatic approach to IAPT service provision. This also needs to be integrated into the already existing IAPT service model and wider services. What lessons learned from past outbreaks can IAPT services implement in order to enhance their response to the mental health aftermath of COVID-19?

## Reflection on responsiveness lessons from past outbreaks

In line with Fig. 2, the aforementioned psychological impact of COVID-19 has highlighted three main affected populations: patients, staff and vulnerable groups. What responsiveness lessons can IAPT learn from past outbreaks in order to respond to the needs of these populations?

### Lessons on meeting the mental health needs of patients

In view of the earlier mentioned mental health impacts on patients, several lessons on service responsiveness applicable to IAPT have been learned from previous outbreaks. For example, in their study on SARS, Mak *et al*. (2009) suggest an integrated multidisciplinary approach to management of complex PTSD cases. This is due to the complex interaction of the biopsychosocial post-pandemic long-term effects. This is in line with the suggestions of other authors, for example, in a summary of the Disaster Mental Health Subcommittee’s observations of the 2009 Swine Flu pandemic, Pfefferbaum *et al*. (2012) highlighted lessons on the importance of an integrated approach to mental and behavioural issues of outbreaks. They also emphasised psychological interventions which focused on addressing fear of uncertainty, enhancing resilience and fostering adaptive behaviours. They also highlighted lessons on the need for public education about how people’s mental health and behaviour might be affected by outbreaks and sources of help. Similarly, in their review article on pandemics, Morganstein *et al*. (2017) summarised lessons on the main areas for long-term outbreak interventions. These include identification of undesirable health-risk behaviours and educating communities on how to build resilience and adapt desirable health-risk behaviours. It also includes managing misinformation and stigma. These findings are comparable to those outlined in the Missouri Department of Health and Senior Services Psychosocial Services preparedness framework for pandemic influenza (2020). This framework emphasises lessons on meeting patients’ mental health needs including tackling anxiety and fear of uncertainty and PTSD. They also emphasise educating the public and managing misinformation, MDT working, involving communities and development of telemental health. ‘Telemental health’ (e.g. digital therapy, e-therapy) is the application of information and communications technologies including telephone, online courses and videoconferencing, to deliver mental health care remotely, including medical consultations and psychotherapy (Waugh *et al*., 2015).

In line with the above lessons on responding to patient needs, some IAPT services adapted quickly to mitigate patient anxieties and fear of uncertainty. They adapted the stepped-care model using wellbeing workshops followed by intervention if needed (e.g. BSMFT, 2020). Many IAPT services have also been adapting their way of working under social distancing and lockdown measures during the acute phase of COVID-19 including remote working via telemental health (e.g. ELFT, 2020). As lockdown eases, services have now started preparing for the aftermath of COVID-19. For example, some services have laid out clear integrated system response plans to build capacity, involve communities, apply digital therapies and reduce inequalities (e.g. ELHCP, 2020). Another example of an integrated MDT approach to mental health is demonstrated by the new Positive Minds mental health service. This provision is a partnership between the local NHS, CCG, City Council, and the voluntary, community and social enterprise sector (PCCG, 2020). The aim of this way of working is to offer a flexible and comprehensive service. These are just a few examples of services across the country. However, anecdotal evidence suggests that most IAPT services are now working to optimise their way of working in the context of COVID-19. In line with the above lessons, and the anticipated significance of PTSD cases, some services have started to prepare for treatment models. For example, Murray *et al*. (2020) have recently adapted a CBT protocol for treating patients with PTSD following ICU. This protocol has adapted the Ehlers and Clark (2000) cognitive model of PTSD so that it can be applied to post-ICU PTSD. This model has reflected on the trauma processes relevant to ICU in relation to COVID-19 and outlines how the PTSD model can be adapted. This protocol has also included provisions to carry out treatment remotely. They also point out the applicability of this protocol to other traumas. Another point for consideration for less complex traumas might be the recently published group TF-CBT protocol for homogeneous traumas (Skilbeck and Spanton, 2020). This group therapy might be beneficial in increasing surge capacity and enhancing normalisation.

### Lessons on meeting the mental health needs of staff

Several lessons on mental health service responsiveness to the needs of staff have been learned from past outbreaks. For example, the Ebola Psychological Support Service (EPSS) for health-care staff was responsively developed during the EVD outbreak (Kamara *et al*., 2017; Waterman *et al*., 2018; Waterman *et al*., 2019). This was a stepped-care approach consisting of several provisions. These included psychoeducation, group wellbeing workshops and individual therapy interventions that were supported by strong leadership. Similarly Maunder *et al*. (2010) described the benefits of computer-assisted resilience training for healthcare worker preparedness for flu pandemics. The programme consisted of several modules including psychoeducation, relaxation, self-assessment and resilience building. This resilience training approach for healthcare workers was also reported by Aiello *et al*. (2011). They used face-to-face group training sessions with a focus on stressors as well as approaches to building resilience and enhancing stress management as facilitated. Another beneficial lesson from the past was from the SARS outbreak. In this case, a Toronto hospital applied staff drop-in psychological support sessions (Maunder *et al*., 2003). They also recommended remote psychological support such as phone or Skype, especially where there was a need to have as few people on site as possible.

In line with Fig. 1, lessons from these past outbreaks have been implemented for the mental health needs of staff in the UK NHS during the acute phase of COVID-19. One example is the proactive ‘nip it in the bud’ Nightingale Hospital Plan (Greenberg, 2020). As outlined in the above lessons, this model emphasises staff psychological first aid focused on recognising the early warning signs of distress in staff and providing support in order to prevent the development of diagnosable mental health disorders whilst also planning for appropriate psychological intervention where needed. These lessons have been further implemented within IAPT service provision. For example, the Homerton Covid Psychological Support (HCPS) pathway delivered by Talk Changes (Hackney & City IAPT) has been developed from the EPSS model (Cole *et al*., 2020). This is a three-phase programme consisting of psychoeducation, low-intensity group therapy and individual CBT interventions. In line with Fig. 1, this model provides an example of how COVID-19 peri-pandemic distress has been addressed for frontline staff using existing resources and infrastructures. The authors anticipate that the application of this model will also be useful in the post pandemic stage of COVID-19. Furthermore, many other IAPT services across England are also offering new pathways to support staff (e.g. Dunn, 2020).

In relation to staff, Mymin Kahn *et al*. (2016) also reported secondary trauma in non-frontline staff in the EVD outbreak. They therefore implemented psychological support for these workers. This lesson from the past is of relevance to IAPT services where IAPT workers may be vulnerable to secondary traumatisation. Therefore, attention must also be paid to staff such as PWPs and therapists working with traumatised COVID-19 cases.

### Lessons on meeting the needs of vulnerable groups

Lessons on responsiveness to the needs of vulnerable groups have been learned from past outbreaks. For example, in their review on pandemics, Morganstein *et al*. (2017) emphasise the importance of identifying vulnerable populations and managing stigma. They also highlight that access issues can also be addressed by considerations around socioeconomic and cultural factors. Similarly, a publication in the *Lancet* on lessons to be learned from the HIV pandemic highlighted the importance of considering health inequalities, in particular older patients and BAME groups (Hargreaves *et al.*, 2020). They emphasise responsive tracking of patient vulnerabilities including socioeconomic status. They advocate means of reaching all patients, especially the hard to reach. They also advise pragmatic approaches that promote behaviour change via MDT interventions and meaningful involvement of communities.

These past lessons have recently been developed for the mental health needs of vulnerable groups in the UK NHS during the acute phase of COVID-19. For example, some IAPT services have been working to identify areas of inequality and need by engaging health and wellbeing boards and local councils to help lead and advise on addressing areas of social inequality (e.g. ELHCP, 2020). There has been consideration for assessments to capture any inequality, for example improving access by identifying digitally excluded populations whilst working within the COVID-19 context. There are also plans to engage patients, e.g. BAME communities and staff, where COVID-19 appears to have had a disproportionate impact and review COVID-19 effects on mental health, health and behaviour change needs. In terms of behaviour change, areas such as anchor programmes, e.g. apprenticeships, have been considered to bridge the gap. Anecdotal evidence suggests that other services have been applying similar models.

In line with Holmes *et al*. (2020), other marginalised groups including those with disabilities and socially excluded groups (e.g. LGBT communities, prisoners and refugees) will also have to be considered. These authors also suggest considerations for people facing job and financial insecurity and digital exclusion. Another lesson from the past from the EVD and HIV outbreaks is that misinformation and irrational fear of contagion can also lead to marginalisation of groups (Davtyan *et al*., 2014).). Therefore, a key response would be considerations for educating communities around the virus.

The key reflections of how IAPT can continue to learn from the past and meet the needs of the above populations (Fig. 2) are summarised in Table 1. In line with the outbreak life-cycle (Fig. 1), this learning should take a pragmatic, continuous and cyclical approach between the phases (Fig. 3). However, as suggested by Holmes *et al*. (2020), there is a need for research to address the short- and long-term mental health effects of COVID-19.

**Table 1.** Summary of the key IAPT responsiveness lessons from past outbreaks

Impact population	Key lessons	Examples of IAPT learning
**Patients**	Working in the context of social distancing	Remote working, e.g. videoconferencing e.g. https://oxcadatresources.com/
	Managing anxiety, stress and PTSD	Online wellbeing workshops, enhancement of existing models, e.g. Murray *et al*. (2020)
	Integrating with communities and wider services	Involvement of communities, councils and social sectors, e.g. ELHCP
**Staff**	Meeting the needs of frontline staff	Timely assessment of need and response, e.g. Greenberg (2020)
	Considerations for non-frontline workers	Considerations for secondary trauma, e.g. Mymin Kahn *et al*. (2016)
	Managing anxiety, stress and PTSD	Resilience building, enhancement of existing models, e.g. Cole *et al*. (2020)
**Vulnerable groups**	Considerations for demographic at-risk factors including age and being of a BAME group	Assessing for differential needs, managing inequalities such as digital exclusion, e.g. ELHCP
	Considerations for other factors such as disability and social exclusion	Involving communities, councils and social sectors, managing stigma through education

## Discussion

As summarised in Table 1, this paper provides a source of reflection on lessons from past outbreaks on how IAPT services can respond to the long-term mental health impacts of COVID-19. Main areas for consideration are taking a pragmatic approach, integrating with other services and responding to community needs. Meeting these needs necessitates community engagement and responding to the needs of staff. With the isolation measures imposed by COVID-19, this paper has highlighted the importance of integrating telemental health into IAPT routine practice. Telemental health is also recommended by the NICE guidelines. For example, in the case of PTSD, they have recommended that there is enough evidence that online and videoconferencing trauma-focused CBT can be as effective as face-to-face therapy (NICE, 2018). Telemental health has been a topic of interest in IAPT services for several years (Thew, 2020). Furthermore, Whaibeh *et al*. (2020) argue that telemental health has demonstrable effectiveness, convenience and reliability, and that it is an essential mode of service provision to be integrated into routine practice. These implementations also integrate with the wider NHS long-term plan. It is imperative that IAPT services continue to adapt a pragmatic approach for non-COVID-19 common mental health problems, including depression, anxiety, panic, OCD and health anxiety, in the context of social distancing measures. Given the undetermined aftermath of COVID-19, this pragmatic and cyclical responsiveness by IAPT will enable it to meet the needs of those affected by COVID-19 whilst also sustaining a wider service.

### Challenges to adaptation

Although IAPT service provision has continued to evolve since its inception, responsiveness to the long-term effects of COVID-19 presents new challenges. These include the fact the pandemic is still ongoing and there are no predictions of its life-cycle and aftermath. Therefore, IAPT services will need to take a flexible approach and plan for surge capacity. Furthermore, adapting to a new ‘normal’ will present challenges for both services and clients. Services will need to ensure that both therapists and clients understand the usefulness of the new ways of service delivery. There may also be financial implications for services setting up additional service delivery models.

### Limitations

To date, research on long-term impacts of pandemics is limited. Furthermore, the presentation of COVID-19 is different from previous pandemics due to its highly contagious nature and undetermined aftermath including long-term physical side-effects. This paper has reflected on lessons on mental health responsiveness from past outbreaks and IAPT implementation. However, there are currently no unitary records of IAPT service responsiveness to the mental health impacts of COVID-19. There is also currently no research evidence as to the expected post-outbreak IAPT referral rates. Therefore, IAPT services would need to monitor the post-COVID-19 trajectory locally as it unfolds. These limitations highlight the importance of a pragmatic approach to IAPT service provision.

## Key practice points

(1) Increasing surge capacity through alternate service delivery modes including telemental health and groups.
(2) Emphasis on the support of patients and staff including IAPT staff who may be prone to secondary traumatisation.
(3) Considerations for vulnerable populations including BAME and hard-to-reach groups.
(4) Adapting treatment models, e.g. for PTSD.
(5) Integrating new ways of working into pre-existing IAPT and wider MDT services.
